# A characterization of some alternating groups *A*_*p*+8_ of degree *p* + 8 by OD

**DOI:** 10.1186/s40064-016-2763-7

**Published:** 2016-07-19

**Authors:** Shitian Liu, Zhanghua Zhang

**Affiliations:** School of Science, Sichuan University of Science and Engineering, Xueyuan Street, Zigong, 643000 Sichuan People’s Republic of China; Sichuan Water Conservancy Vocational College, Chongzhou, Chengdu, 643000 Sichuan People’s Republic of China

**Keywords:** Element order, Alternating group, Simple group, Symmetric group, Degree pattern, Prime graph, 20D05, 20D06, 20D60

## Abstract

Let $A_n$ be an alternating group of degree *n*. We know that $A_{10}$ is 2-fold *OD*-characterizable and $A_{125}$ is 6-fold *OD*-characterizable. In this note, we first show that $A_{189}$ and $A_{147}$ are 14-fold and 7-fold OD-characterizable, respectively, and second show that certain groups $A_{p+8}$ with that $\pi ((p+8)!)=\pi (p!)$ and $p<1000$, are OD-characterizable. The first gives a negative answer to Open Problem of Kogani-Moghaddam and Moghaddamfar.

## Background

For a group, it means finite, and for a simple group, it is non-abelian. If *G* is a group, then the set of element orders of *G* is denoted by $\omega (G)$ and the set of prime divisors of *G* is denoted by $\pi (G)$. Related to the set $\omega (G)$ a graph is named a prime graph of *G*, which is written by *GK*(*G*). The vertex set of *GK*(*G*) is written by $\pi (G)$, and for different primes *p*, *q*, there is an edge between the two vertices *p*, *q* if $p\cdot q\in \omega (G)$, which is written by $p\sim q$. We let *s*(*G*) denote the number of connected components of the prime graph *GK*(*G*).

Moghaddamfar et al in 2005 gave the following notions which inspire some authors’ attention.

### **Definition 1**

(Moghaddamfar et al. 2005) Let *G* be a finite group and $|G|=p_{1}^{\alpha _{1}}p_{2}^{\alpha _{2}}\cdots p_{k}^{\alpha _{k}}$, where $p_{i}$s are primes and $\alpha _{i}$s are positive integers. For $p\in \pi (G)$, let $\deg (p):=|\{q\in \pi (G)|p\sim q\}|$, which we call the degree of *p*. We also define $D(G):=(\deg (p_{1}),\deg (p_{2}),\ldots ,\deg (p_{k}))$, where $p_{1}<p_{2}<\cdots <p_{k}$. We call *D*(*G*) the degree pattern of *G*.

For a given finite group *M*, write $h_{OD}(M)$ to denote the number of isomorphism classes of finite groups *G* such that (1) $|G|=|M|$ and (2) $D(G)=D(M)$.

### **Definition 2**

(Moghaddamfar et al. 2005) A finite group *M* is called *k*-fold OD-characterizable if $h_{OD}(M)=k$. Moreover, a 1-fold OD-characterizable group is simply called an OD-characterizable group.

Up to now, some groups are proved to be *k*-fold OD-characterizable and we can refer to the corresponding references of Akbari and Moghaddamfar (2015).

Concerning the alternating group *G* with $s(G)=1$, what’s the influence of OD on the structure of group? Recently, the following results are given.

### **Theorem 3**

*The following statements hold:*

1. *The alternating group*$A_{10}$*is 2-fold OD-characterizable* (*see* Moghaddamfar and Zokayi 2010).
2. *The alternating group*$A_{125}$*is 6-fold OD-characterizable (see* Liu and Zhang Submitted).
3. *The alternating group*$A_{p+3}$*except*$A_{10}$*is OD-characterizable (see* Hoseini and Moghaddamfar 2010; Kogani-Moghaddam and Moghaddamfar 2012; Liu 2015; Moghaddamfar and Rahbariyan 2011; Moghaddamfar and Zokayi 2009; Yan and Chen 2012; Yan et al. 2013; Zhang and Shi 2008; Mahmoudifar and Khosravi 2015).
4. *All alternating groups*$A_{p+5}$, *where*$p+4$*is a composite and*$p+6$*is a prime and*$5\ne p\in \pi (1000!)$, *are OD-characterizable (see* Yan et al. 2015).

*In* Moghaddamfar (2015), $A_{189}$*is at least 14-fold OD-characterizable. In this paper, we show the results as follows.*

### **Theorem 4**

*The following hold:*

1. *The alternating group*$A_{189}$*of degree 189 is 14-foldOD-characterizable.*
2. *The alternating group*$A_{147}$*of degree 147 is 7-fold OD-characterizable*.

These results give negative answers to the Open Problem (Kogani-Moghaddam and Moghaddamfar 2012).

### **Open Problem**

 (Kogani-Moghaddam and Moghaddamfar 2012) All alternating groups $A_m$, with $m \ne 10$, are OD-characterizable.

We also prove that some alternating groups $A_{p+8}$ with $p<1000$ are OD-characterizable.

### **Theorem 5**

*Assume thatpis a prime satisfying the following three conditions:*

1. $$p\ne 139$$*and*$$p\ne 181$$,
2. $$\pi ((p+8)!)=\pi (p!)$$,
3. $$p\le 997$$.

*Then the alternating group*$A_{p+8}$*of degree*$p+8$*is OD-characterizable*.

Let *G* be a finite group, then let $\mathrm {Soc}(G)$ denote the socle of *G* regarded as a subgroup which is generated by the minimal normal subgroup of *G*. Let $\mathrm {Syl}_{p}(G)$ be the set of all Sylow *p*-subgroups $G_p$ of *G*, where $p\in \pi (G)$. Let $\mathrm {Aut}(G)$ and $\mathrm {Out}(G)$ be the automorphism and outer-automorphism group of *G*, respectively. Let $S_{n}$ denote the symmetric groups of degree *n*. Let *p* be a prime divisor of a positive integer *n*, then the *p*-part of *n* is denoted by $n_p$, namely, $n_p\Vert n$. The other symbols are standard (see Conway et al. 1985, for instance).

## Some preliminary results

In this section, some preliminary results are given to prove the main theorem.

### **Lemma 6**

*Let*$S=P_1\times \cdots \times P_r$, *where*$P_i$’*s are isomorphic non-abelian simple groups. Then*$\mathrm {Aut}(S)=\mathrm {Aut}(P_1)\times \cdots \times \mathrm {Aut}(P_r).S_r$.

### *Proof*

See Zavarnitsin (2000). $$\square$$

### **Lemma 7**

*Let*$A_{n}$ (*or*$S_{n}$) *be an alternating (or a symmetric group) of degreen*. *Then the following hold.*

1. *Let*$p,q\in \pi (A_{n})$*be odd primes. Then*$p\sim q$*if and only if*$p+q\le n$.
2. *Let*$p\in \pi (A_{n})$*be odd prime. Then*$2\sim p$*if and only if*$p+4\le n$.
3. *Let*$p,q\in \pi (S_{n})$. *Then*$p\sim q$*if and only if*$p+q\le n$.

### *Proof*

It is easy to get from Zavarnitsin and Mazurov (1999). $\square$

### **Lemma 8**

*The number of groups of order 189 is 13.*

### *Proof*

See Western (1898). $$\square$$

### **Lemma 9**

*LetPbe a finite simple group and assume thatris the largest prime divisor of* |*P*| *with*$50< r<1000$. *Then for every prime numberssatisfying the inequality*$(r - 1)/2 < s \le r$, *the order of the factor group*$\mathrm {Aut}(P)/P$*is not divisible bys*.

### *Proof*

It is easy to check this results by Conway et al. (1985) and Zavarnitsine (2009). $\square$

Let $n=p_1^{\alpha _1}p_2^{\alpha _2}\cdots p_r^{\alpha _r}$ where $p_1,p_2,\ldots , p_r$ are different primes and $\alpha _1,\alpha _2,\ldots ,\alpha _r$ are positive integers, then $\exp (n,p_i)=\alpha _i$ with $p_{i}^{\alpha _i}\mid n$ but $p_i^{\alpha _i+1}\nmid n$.

### **Lemma 10**

*Let*$L:=A_{p+8}$*be an alternating group of degree*$p+8$*with thatpis a prime and*$\pi (p+8)!=\pi (p!)$. *Let*$|\pi (A_{p+8})|=d$*withda positive integer. Then the following hold:*

1. $$\deg (p)=4$$*and*$$\deg (r)=d-1$$*for*$$r\in \{2,3,5,7\}$$.
2. $$\exp (|L|,2)\le p+7$$.
3. $\exp (|L|,r)=\sum \nolimits _{i=1}^{\infty }[\frac{p+8}{r^i}]$*for each*$r\in \pi (L)\backslash \{2\}$. *Furthermore,*$\exp (|L|,r)<\frac{p+8}{2}$ where $5\le r\in \pi (L)$. *In particular, if*$r>[\frac{p+8}{2}]$, *then*$\exp (|L|,r)=1$.

### *Proof*

By Lemma 7, it is easy to compute that for odd prime $r, p\cdot r\in \omega (L)$ if and only if $p+r\le p+8$. Hence $r=3,5,7$. If $r=2$, then since $p+4\le p+8$, then $2\cdot p\in \omega (L)$. This completes (1).

By Gaussian’s integer function,

$$\begin{aligned} \exp (|L|,2)&= \sum \limits _{i=1}^{\infty }\left[ \frac{p+8}{2^i}\right] -1\\&= \left( \left[ \frac{p+8}{2}\right] +\frac{p+8}{2^2}+ \left[ \frac{p+8}{2^3}\right] +\cdots \right) -1\\&\le \left( \frac{p+8}{2}+\frac{p+8}{2^2}+\frac{p+8}{2^3}+\cdots \right) -1\\&= p+7. \end{aligned}$$

This proves (2). Similarly, we can get (3). $\square$

### **Lemma 11**

*Leta*, *mbe positive integers. If*$(a,m)=1$, *then the equation*$a^x\equiv 1$(*modm*) *has solutions. In particular, if the order of a modulomish*(*a*), *thenh*(*a*) *divides*$\phi (m)$*where*$\phi (m)$*denotes the Euler’s function ofm*.

### *Proof*

See Theorem 8.12 of Burton (2002).$\square$

### **Lemma 12**

*Letpbe a prime and*$L:=A_{p+8}$*be the alternating group of degree*$p+8$*with that*$\pi ((p+8)!)=\pi (p!)$. *Given*$P\in \mathrm {Syl}_p(L)$*and*$Q\in \mathrm {Syl}_q(L)$*with*$11\le q<p\le 1000$. *Then the following results hold:*

1. *The order of*$N_L(P)$*is not divisible by*$q^{s(q)}$, *where*$s(q)=\exp (|L|,q)$.
2. *If*$p\in \{113, 139, 199, 211, 241, 283, 293, 337, 467, 509, 619, 787, 797, 839, 863, 887, 953, 997\}$, *then*$|N_L(Q)|$*is not divisible byp*.
3. *If*$p\in \{181, 317, 409, 421, 523, 547, 577, 631, 661, 691, 709, 811, 829, 919\}$, *then there is at least a primerwith that the order of r modulopis less than*$p-1$, *where*$11\le r<p$*and*$r\in \pi (p!)$.

### *Proof*

By Lemma 11, the equation $q^x\equiv 1$(mod *p*) has solutions. Suppose the order of *q* modulo *p* is written by *h*(*q*). If $h(q)=p-1$, then *q* is a primitive root of modulo *p*. By Lemma 11, we have $h(q)\mid p-1$. By Lemma 10, we can get *s*(*q*). If $h(q)>s(q)$, then $q^{h(q)}\mid |L|$, a contradiction to the hypotheses. Then we can assume that $h(q)\le s(q)$. We can get the *q* and *h*(*q*) by GAP (2016) as Table 1 (Note that there is certain prime which has order $h(q)<p-1$, but $h(q)>s(q)$. Hence we do not list in this table).

By NC Theorem, the factor group $\frac{N_L(P)}{C_L(P)}$ is isomorphic to a subgroup of $\mathrm {Aut}(P)\cong \mathbb {Z}_{p-1}$ where $\mathbb {Z}_n$ is a cyclic group of order *n*. It follows that the order of $\frac{N_L(P)}{C_L(P)}$ is less than or equal to $p-1$. If $11\le q<p$ and $q^{s(q)}\mid |N_L(P)|$ where $\exp (|L|,q)=s(q)$, then $q\mid |C_L(P)|$. This forces $q\sim p$, a contradiction. This ends the proof of (1).

Next, assume that $p\in \{ 113, 139, 199, 211, 241, 283, 293, 337, 467, 509, 619, 787, 797, 839, 863, 887, 953, 997\}$. If *p* divides the order of $N_L(Q)$, then by NC theorem and Table 1, $p\mid |C_L(Q)|$ and so $p\sim q$, a contradiction. This proves (2). (3) follows from Table 1.

This completes the proof of Lemma 12. $\square$

**Table 1:** The values of *p* and *h*(*q*)

*p*	*h*(*q*)	Condition	*p*	*h*(*q*)	Condition
113	$2^4.7$	None	139	2.3.23	None
181	$2^2.3^2.5$	$q\ne 19$	181	4	$q=19$
199	$2.3^2.11$	None	211	2.3.5.7	None
241	$2^4.3.5$	None	283	2.3.47	None
293	$2^2.73$	None	317	$2^2.79$	$q\ne 73$
317	4	$q=73$	337	$2^4.3.7$	None
409	$2^3.3.17$	$q\ne 31,53$	409	8	$q=31$
409	3	$q=53$	421	$2^2.3.5.7$	$q\ne 29$
421	4	$q=29$	467	2.233	None
509	$2^2.127$	none	523	$2.3^2.29$	$q\ne 11,19,61$
523	29	$q=11$	523	9	$q=19$
523	6	$q=61$	547	2.3.7.13	$q\ne 11,13,41$
547	39	$q=11$	547	21	$q=13$
547	6	$q=41$	577	$2^6.3^2$	$q\ne 23$
577	8	$q=23$	619	2.3.103	None
631	$2.3^2.5.7$	$q\ne 43$	631	3	$q=43$
661	$2^3.3.5.11$	$q\ne 11$	661	33	$q=11$
691	2.3.5.23	$q\ne 89$	691	5	$q=89$
709	$2^2.3.59$	$q\ne 227$	709	3	$q=227$
787	2.3.131	None	797	$2^2.199$	None
811	$2.3^4.5$	$q\ne 131$	811	6	$q=131$
829	$2^2.3^2.23$	$q\ne 11$	829	23	$q=11$
839	2.419	None	863	2.431	None
887	2.443	None	919	$2.3^3.17$	$q\ne 53$
919	6	$q=53$	953	$2^3.7.17$	None
997	$2^2.3.83$	None			

## Proof of the main theorem

In this section, we first give the proof of Theorem 4 and second prove Theorem 5.

### The proof of Theorem 4

#### *Proof*

We divides the proof into two steps.

***Step 1*** Let $M=A_{189}$. Assume that *G* is a finite group such that

$$|G|=|M|$$

and

$$D(G)=D(M).$$

By Lemma 7, the degree pattern *GK*(*G*) of *G* is connected, in particular, the degree pattern *GK*(*G*) is the same as the degree pattern of *GK*(*M*).

#### **Lemma 13**

*LetKbe a maximal normal soluble subgroup ofG*. *ThenKis a*$\{2, 3, 5, 7\}$-*group, in particular*, *Gis insoluble*.

#### *Proof*

Assume the contrary. First we show that *K* is a $181'$-group. We assume that *K* contains an element *x* of order 181. Let *C* be the centralizer of *x* in *G* and *N* be the normalizer of *x* in *G*. It is easy to see from *D*(*G*) that *C* is a $\{2, 3, 5, 7, 181\}$-group. By NC theorem, *N*/*C* is isomorphic to a subgroup of automorphism group $\mathrm {Aut}(\langle x\rangle)\cong \mathbb {Z}_{2^2}\times \mathbb {Z}_{3^2}\times \mathbb {Z}_5$, where $\mathbb {Z}_n$ is a cyclic group of order *n*. Hence, *C* is a $\{2, 3, 5, 7, 181\}$-group. By Frattini’s arguments, $G=KN_G(\langle x\rangle)$ and so $\{11, 13, 17, 19, 23, 29, 31, 37, 41, 43, 47, 53, 59, 61, 67, 71, 73, 79, 83, 89, 97, 101, 103, 107, 109, 113, 127, 131, 137, 139, 149, 151, 157, 163, 167, 173, 179, 181\}\subseteq \pi (K)$. Since *K* is soluble, *G* has a Hall subgroup *H* of order $109\cdot 181$. Obviously, $109\nmid 181-1, H$ is cyclic and so $109\cdot 181\in \omega (G)$ contradicting $D(G)=D(M)$.

Second, show that *K* is a $p'$-group, where $p\in \{11, 13, 17, 19, 23, 29, 31, 37, 41, 43, 47, 53, 59, 61, 67, 71, 73, 79, 83, 89, 97, 101, 103, 107, 109, 113, 127, 131, 137, 139, 149, 151, 157, 163, 167, 173, 179\}$. Let *p* be a prime divisor of |*K*| and *P* a Sylow *p*-subgroup of *K*. By Frattini’s arguments, $G=KN_G(P)$. It follows from Lemma 12, that 181 is a divisor of $|N_G(P)|$ if and only if $p=19$. If $181\nmid |\mathrm {Aut}(P)|$, then 181 divides the order of $C_G(P)$ and so there is an element of order $p\cdot 181$, a contradiction. On the other hand, $p=19$ and $181\mid |\mathrm {Aut}(P)|$, where *P* is the Sylow 19-subgroup of *K*. By Lemma 10, $\exp (|L|,19)=9$ and so $|\frac{N_G(P)}{C_G(P)}|\mid \prod \nolimits _{i=1}^{9}19^{45}\cdot (19^i-1)$. It is easy to get that $101\nmid \mid \prod \nolimits _{i=1}^{9}19^{45}\cdot (19^i-1)$. If $101\mid |N_G(P)|$, then 101 is a prime divisor of $C_G(P)$. Set $C=C_G(P)$ and $C_{101}\in \mathrm {Syl}_{101}(C)$. Also $\exp (|L|,101)=1$. By Frattini’s argument, $N=CN_N(C_{101})$ and so $p\nmid |N_N(C_{101})|$. Thus $181\mid |C|$ and so $181\sim p$, a contradiction. So $101\nmid |N_G(P)|$ and $101\in \pi (K)$. Let $K_{101}\in \mathrm {Syl}_{101}(K)$. Since $G=KN_G(K_{101})$, 101 divides the order of $N_G(K_{101})$, then $101\nmid |K|$, a contradiction. Therefore *K* is a $\{2,3,5,7\}$-group.

Obviously, $G\ne K$ and so *G* is insoluble. $\square$

#### **Lemma 14**

*The quotient groupG* / *Kis an almost simple group. More precisely, there is a normal series such that*$S\le G/K\le \mathrm {Aut}(S)$, *whereSis isomorphic to*$A_{n}$*for*$n\in \{181, 182, 183, 184, 185, 186, 187, 188, 189\}$.

#### *Proof*

Let $H=G/K$ and $S=\mathrm {Soc}(H)$. Then $S=B_1\times \cdots \times B_n$, where $B_i$’s are non-abelian simple groups and $S\le H\le \mathrm {Aut}(S)$. In what follows, we will prove that $n=1$ and $S\cong A_{n}$.

Suppose the contrary. Obviously, 181 does not divide the order of *S*, otherwise, there is an element of order $109\cdot 181$ contradicting $D(G)=D(A_{189})$. Hence, for every *i*, we have that $B_i\in \mathfrak {F}_{179}$, where $\mathfrak {F}_p$ is the set of non-abelian simple group *S* with that $p\in \pi (S)\subseteq \{2,3,\cdots ,p\}$ and *p* is a prime. But by Lemma 13, *K* is a $\{2,3,5,7\}$-group. Therefore $181\in \pi (H)\subseteq \pi (\mathrm {Aut}(S))$ and so 181 divides the order of $\mathrm {Out}(S)$. By Lemma 6, $\mathrm {Out}(S)=\mathrm {Out}(P_1)\times \cdots \times \mathrm {Out}(P_r)$, where the group $P_i$’s are satisfying $S\cong P_1\times \cdots \times P_r$. Therefore for some *j* 181 divides the order of an outer-automorphism group of a direct $P_j$ of *t* isomorphic simple group $B_i$. Since $B_i\in \mathfrak {F}_{179}$, the order of $\mathrm {Out}(B_i)$ is not divisible by 181 by Lemma 9. By Lemma 6, $|\mathrm {Aut}(P_j)|=|\mathrm {Aut}(P_j)|^t\cdot t!$. It means $t\ge 181$, and hence $4^{181}\mid |G|$, a contradiction. Thus $n=1$ and $S=B_1$.

By Lemma 13, we can assume that $|S|=2^a\cdot 3^b\cdot 5^c\cdot 7^{d}\cdot 11^{18}\cdot 13^{15}\cdot 17^{11}\cdot 19^9\cdot 23^8\cdot 29^6\cdot 31^6\cdot 37^5\cdot 41^4\cdot 43^4\cdot 47^4\cdot 53^4\cdot 59^3\cdot 61^3\cdot 67^2\cdot 71^2\cdot 73^2\cdot 79^2\cdot 83^2\cdot 89^2\cdot 97\cdot 101\cdot 107\cdot 109\cdot 113\cdot 127\cdot 131\cdot 137\cdot 139\cdot 149\cdot 151\cdot 157\cdot 163\cdot 167\cdot 173\cdot 179\cdot 181$, where $2\le a \le 182, 1\le b\le 93, 1\le c\le 45$ and $1\le d\le 30$. By Zavarnitsine (2009), the only possible group is isomorphic to $A_n$ with $n\in \{181, 182, \ldots , 189\}$.

This completes the proof. $\square$

We continue the proof of Theorem 4. By Lemma 14, *S* is isomorphic to $A_{n}$ with $n\in \{181, 182, \cdots , 189\}$, and $S\le G/K\le \mathrm {Aut}(S)$.

#### *Case 1*

Let $$S\cong A_{181}$$.

Then $A_{181}\le G/K\le S_{181}$. If $G/K\cong A_{181}$, then $|K|=182\cdot 183\cdot 184\cdot 185\cdot 186\cdot 187\cdot 188\cdot 189=2^6\cdot 3^5\cdot 5\cdot 7^2\cdot 11\cdot 13\cdot 17\cdot 23\cdot 31\cdot 37\cdot 47$ and so $11,13,17,23,31,37,47\in \pi (K)$ contradicting to Lemma 13.

If $G/K\cong S_{181}$, we also have that 11, 13, 17 or 19 divides |*K*|, contradicting to Lemma 13.

Similarly we can rule out these cases “$S\cong A_{n}$ with $n\in \{182, 183, \cdots , 187\}$”.

#### *Case 2*

Let $$S\cong A_{188}$$.

Then $A_{188}\le G/K\le S_{188}$. Therefore $G/K\cong A_{188}$ or $G/K\cong S_{188}$.

(1.1) Let $G/K\cong A_{188}$. Then $|K|=7\cdot 3^3$. By Conway et al. (1985), the order of $\mathrm {Out}(A_{188})$ is 2 and the Schur multiplier of $A_{188}$ is 2. Then *G* is isomorphic to $K\times A_{188}$. By Lemma 8, there are 13 types of groups of order 189 satisfying that $|G|=|M|$ and $D(G)=D(M)$.
(1.2) Let $G/K\cong S_{188}$. Since $|S_{188}|_2=|S_{189}|_2>|A_{189}|_2$, then we rule out this case.

#### *Case 3*

 Let $$S\cong A_{189}$$.

Then $A_{189}\le G/K\le S_{189}$. If $G/K\cong A_{189}$, then order consideration implies that *G* is isomorphic to $A_{189}$. If $G/K\cong S_{189}$, then as $|S_{189}|_2>|A_{189}|_2=|G|_2$, we rule out this case.

***Step 2*** Similarly as the proof of (1), the following results are given:

1. *K* is a maximal soluble normal $\{2,3,5,7\}$-group.
2. $S\le G/K\le \mathrm {Aut}(S)$, where *S* is isomorphic to one of the groups: $A_{139}, A_{140}, \ldots , A_{146}$ and $A_{147}$.

#### *Case 1*

Let $$S\cong A_{139}$$.

Then $A_{139}\le G/K\le S_{139}$. If the former, then $11\mid |K|$, a contradiction. If the latter, we also have that $11\mid |K|$ and so we rule out.

Similarly we can rule out these cases “*S* is isomorphic to $A_{140}, A_{141}, \ldots , A_{145}$”.

#### *Case 2*

 Let $$S\cong A_{146}$$.

Then $A_{146}\le G/K\le S_{146}$. If $G/K\cong A_{146}$, then $|K|=3\cdot 7^2$. Since the order of $\mathrm {Out}(A_{147})$ is 2 and the Schur multiplier of $A_{147}$ is 2. Then *G* is isomorphic to $K\times A_{146}$. By GAP (2016), there are six types of groups of order 147. So there are 6 groups with the hypotheses: $|G|=|A_{147}|$ and $D(G)=D(A_{147})$. If $G/K\cong S_{147}$, then as $|S_{146}|_2>|A_{146}|_2=|A_{147}|_2=|G|_2$, we rule out.

#### *Case 3*

 Let $$S\cong A_{147}$$.

Then $A_{147}\le G/K\le S_{147}$. If the former, then $K=1$ and so $G\cong A_{147}$, the desired result. If the latter, then as $|S_{147}|_2>|A_{147}|_2=|G|_2$, we rule out.

We also can get that $A_{147}$ is 7-fold OD-characterizable.

This completes the proof of Theorem 4. $\square$

### The proof of Theorem 5

#### *Proof*

Assume that $|G|=|A_{p+8}|$ and $D(G)=D(A_{p+8})$, then by Lemma 7, the degree pattern *GK*(*G*) of *G* is the same as $GK(A_{p+8})$ of $A_{p+8}$. Similarly as the proof of Theorem 4, the statements are gotten:

1. Let *K* be a maximal soluble group. Then *K* is a $\{2, 3, 5, 7\}$-group, in particular, *G* is insoluble.
2. There is a normal series such that $S\le G/K\le \mathrm {Aut}(S)$, where *S* is isomorphic to $A_{p+r}$ with that $0\le r\le 8$ and $p\in \{$113, 139, 199, 211, 241, 283, 293, 317, 337, 409, 421, 467, 509, 523, 547, 577, 619, 631, 661, 691, 709, 787, 797, 811, 829, 839, 863, 887, 919, 953, 997$\}$.

In what follows, we consider the case “$p=113$”.

1. $$S\cong A_{113}$$.
  Then $A_{113}\le G/K\le S_{113}$. If $G/K\cong A_{113}$, then 11 divides the order of *K*, a contradiction. If $G/K\cong S_{113}$, then we also have that $11\mid |K|$, a contradiction. Similarly we can get a contradiction when *S* is isomorphic to one of $A_{114}, A_{115}, A_{116}, A_{117}, A_{118}, A_{119}$, and $A_{120}$.
2. Let $$S\cong A_{121}$$.
  Then $A_{121}\le G/K\cong S_{121}$. If $G/K\cong A_{121}$, then $K=1$, the desired result. If $G/K\cong S_{121}$, then as $|S_{121}|_2>|G|_2=|A_{121}|_2$, a contradiction.

Similarly we can deal with these cases “$p\in \{139, 199, 211, 241, 283, 293, 317, 337, 409, 421, 467, 509, 523, 547, 577, 619, 631, 661, 691, 709, 787, 797, 811, 829, 839, 863, 887, 919, 953, 997\}$”.

This completes the proof of Theorem 5. $\square$

## Non OD-characterization of some alternating groups

Assume that *p* is a prime and *m* is an integer larger than 3. If $\pi ((p+m)!)\subseteq \pi (p!)$, then $GK(A_{p+m})$ is connected.

For the alternating group $A_{p+m}, |A_{p+m}|=(p+m)|A_{p+m-1}|$.

We shall use the notation *v*(*n*) to denote the number of types of groups of order *n* where *n* is a positive integer. We follows the method of Moghaddamfar (2015), $h_{OD}(A_{p+m})\ge 1+v(p+m)$ where $\pi (A_{p+m})=\pi (A_p)$ and $m\ge 1$ is a non-prime integer. We get the results as Table 2 which contains some results of Liu and Zhang (Submitted), Moghaddamfar (2015), Mahmoufifar and Khosravi (2014).

**Table 2:** Non OD-characterizability of alternating groups

*G*	*p*	*m*!	$\pi (p+m)$	$h_{OD}$	References
$A_{125}$	113	12!	$\{5\}$	≥6	Mahmoufifar and Khosravi (2014)
$A_{147}$	139	8!	$\{3,7\}$	≥7	Moghaddamfar (2015)
$A_{189}$	181	8!	$\{3,7\}$	≥14	Moghaddamfar (2015)
$A_{539}$	523	16!	$\{7,11\}$	≥3	Moghaddamfar (2015)
$A_{625}$	619	6!	$\{5\}$	≥16	Moghaddamfar (2015)
$A_{875}$	863	12!	$\{13,67\}$	≥6	Moghaddamfar (2015)
$A_{1029}$	1019	10!	$\{3,7\}$	≥20	
$A_{1144}$	1129	15!	$\{2,11,13\}$	≥40	
$A_{1274}$	1159	15!	$\{2,7,13\}$	≥11	
$A_{1344}$	1319	25!	$\{2,3,7\}$	≥11,721	
$A_{1352}$	1319	33!	$\{2,13\}$	≥53	

Note that *v*(*n*), the number of groups of given small order *n* can be computed by GAP (2016). The Gap programme is as followings.

gap> SmallGroupsInformation(n);

So we have the following conjecture.

**Conjecture** Assume that *p* is a prime and $m\ge 6$ is not a prime. If $\pi ((p+m)!)\subseteq \pi (p!)$ and $\pi (p+m)\subseteq \pi (m!)$, then $A_{p+m}$ is not OD-characterizable.

## Conclusion

In this paper, we have proved the following two results.

**Result 1a:** The alternating group $A_{189}$ of degree 189 is 14-fold OD-characterizable.

**Result 1b:** The alternating group $A_{147}$ of degree 147 is 7-fold OD-characterizable.

**Result 2:** Let *p* be a prime with the following three conditions:

1. $$p\ne 139$$ and $$p\ne 181$$,
2. $$\pi ((p+8)!)=\pi (p!)$$,
3. $$p\le 997$$.

Then the alternating group $A_{p+8}$ of degree $p+8$ is OD-characterizable.
